# Evaluating Changes in Pulsatile Flow With Endovascular Stents in an In Vitro Blood Vessel Model: Potential Implications for the Future Management of Neurovascular Compression Syndromes

**DOI:** 10.7759/cureus.59811

**Published:** 2024-05-07

**Authors:** Imran Siddiqi, James Brazdzionis, Jordan M Hough, Louis Reier, Maxwell Marino, Katherine Ko, Michael Schiraldi, Vladimir Cortez, Dan E Miulli

**Affiliations:** 1 Neurosurgery, Riverside University Health System Medical Center, Moreno Valley, USA; 2 Neurosurgery, Arrowhead Regional Medical Center, Colton, USA; 3 Neurosurgery, California University of Science and Medicine, Colton, USA

**Keywords:** pulsatile flow, glossopharyngeal neuralgia, pulsatility, neurovascular compression, hemifacial spasms, endovascular stenting, trigeminal neuralgia

## Abstract

Background: Neurovascular compression syndromes (NVCS), encompassing conditions such as trigeminal neuralgia, hemifacial spasm, and glossopharyngeal neuralgia, significantly impair patient quality of life through abnormal vascular compression and micro-pulsation of vasculature on cranial nerves at the Obersteiner-Redlich zone. The modulation of pulsatile flow dynamics via endovascular stents presents a novel research frontier for alleviating these syndromes.

Aim: The primary aim of this investigation was to delineate the impact of various endovascular stents on pulsatile flow within an in vitro model of a blood vessel, thereby elucidating their potential applicability in the therapeutic management of NVCS.

Materials and methods: A simple in vitro analog of a posterior circulation artery was developed, employing an intravenous pump to replicate cardiac-induced blood flow. Within this model, alterations in pulsatile flow were quantitatively assessed following the introduction of three categorically distinct endovascular stents, varying in size. This assessment was facilitated through the employment of both micro-Doppler and Doppler ultrasound methodologies.

Results: The Pipeline 5x35 mm stent (Medtronic, Minneapolis, MN) demonstrated the most significant reductions in peak systolic velocity (Vmax) and pulsatility index (PI), PI especially over the stent, suggesting its potential for drastically altering blood flow dynamics. Similarly, Neuroform Atlas 4.5x30 mm and Neuroform Atlas 4x24 mm stents (Stryker, Kalamazoo, MI) also showed notable decreases in hemodynamic parameters, albeit to different extents. Statistical analysis confirmed that these changes were significantly different from the control (P < 0.0001 for PI and Vmax; P < 0.05 for inter-stent comparisons), except for proximal PI means, which did not significantly differ from the control (P = 0.2777).

Conclusion: These findings affirm the potential of endovascular stents to substantially modulate arterial pulsatility. The observed decrease in pulsatile flow resultant from endovascular stent application has the potential to attenuate ectopic nerve excitation, a hallmark of NVCS. Consequently, this research highlights the prospective utility of endovascular stents in developing minimally invasive therapeutic approaches for NVCS.

## Introduction

Neurovascular compression syndromes (NVCS), encompassing conditions such as trigeminal neuralgia (TN), hemifacial spasm (HFS), and glossopharyngeal neuralgia, represent a significant clinical challenge, primarily arising from the aberrant vascular compression of cranial nerves at their root entry or exit zones (REZ) from the brainstem [1]. Among these, TN and HFS are notably prevalent, causing considerable morbidity and impacting patient quality of life [2]. TN, characterized by neuropathic facial pain manifesting as sudden, unilateral, sharp, and electric shock-like sensations along the trigeminal nerve’s distribution, is acknowledged as one of the most severe neurological disorders [2]. It leads to significant functional impairment, categorized into classic, resulting from direct vascular compression of the trigeminal nerve root with injury to the nerve from micro-pulsations; secondary, associated with a broader neurological disorder; and idiopathic forms. Conversely, HFS is delineated by episodic, involuntary contractions of facial muscles on one side, attributed to abnormal activation of the facial nerve, often resulting from vascular compression [3]. The pathophysiological underpinnings for both classic and secondary forms of these conditions include focal demyelination of primary afferents near the trigeminal root's entry into the pons, predisposing the axons to hyperexcitability, ectopic excitation, and high-frequency discharges [4]. This ectopic nerve activity, potentially triggered by arterial pulsation, underscores most TN cases, often attributed to compression by adjacent arteries, with the superior cerebellar artery (SCA) being most commonly implicated in TN. Microvascular decompression surgery has provided immediate relief by mechanically separating the offending artery from the nerve root, thus mitigating the pulsatile mechanical stimulation at the REZ initiating ephaptic transmission, believed to contribute to these disorders [5]. However, this procedure, despite its efficacy, carries risks and limitations, emphasizing the need for less invasive treatment modalities.

Recent advances in endovascular technology, including the development of stents and flow diverters, have introduced new potential therapeutic avenues [6]. These devices, designed to modify vascular dynamics, may offer a minimally invasive alternative to traditional surgery, potentially reducing ectopic nerve excitation by altering arterial pulsatility [7]. Through a novel in vitro model simulating the arterial conditions implicated in NVCS, this study aims to provide empirical evidence supporting the potential efficacy of stent deployment in reducing pulsatile flow and, by extension, ectopic nerve activity associated with TN and HFS.

NVCS such as TN, HFS, and glossopharyngeal neuralgia are challenging conditions often stemming from the vascular compression of cranial nerves at their REZ from the brainstem. TN presents as neuropathic facial pain, typically characterized by sudden, one-sided, sharp, electric shock-like pain that follows the trigeminal nerve's path across the forehead, cheek, and lower jaw [8]. Recognized as one of the most excruciating neurological disorders, it can lead to substantial impairment [9]. HFS, on the other hand, involves episodic, involuntary twitching of facial muscles on one side, served by the corresponding facial nerve (seventh cranial nerve). This condition is characterized by the abnormal, involuntary activation of the peripheral facial nerve, causing brief or prolonged contractions in the facial expression muscles [10]. In both classic and secondary TN, it is theorized that the primary issue is the focal demyelination of the primary trigeminal afferents near where the trigeminal root enters the pons. This demyelination makes the axons more prone to hyperexcitability and increases their susceptibility to ectopic excitation and high-frequency discharges [4]. Ectopic activity in the nerve can be spontaneous or triggered by direct mechanical stimulation, such as arterial pulsation. The majority of TN cases are attributed to compression of the trigeminal nerve root near its entry into the pons [4]. In primary HFS, the underlying pathophysiological mechanism is similar to that of primary TN, but it involves compression of the facial nerve, typically by the anterior inferior cerebellar artery (AICA), vertebral artery (VA), or posterior inferior cerebellar artery [11]. This mechanism explains the often immediate relief of pain and cessation of facial spasms following microvascular decompression surgery. In this procedure, utilized for both TN and HFS, a synthetic Teflon material is used to separate the artery from the nerve root, thereby mitigating the effects of arterial pulsation on the trigeminal or facial nerve [5]. During the assessment of patients, neuroimaging techniques such as high-resolution brain MRI are crucial in identifying vascular compression and in detecting secondary lesions like aneurysms or tumors.

Current treatments for neurovascular compression syndromes

For both types of NVCS, carbamazepine or oxcarbazepine is often the initial treatment of choice. However, its effectiveness can decrease over time for some patients, and it may cause undesirable side effects, including drowsiness, dizziness, double vision, and nausea [12]. Alternative treatments can include anticonvulsants like lamotrigine, phenytoin, gabapentin, and botulinum toxin injections [13,14]. If these medical treatments prove ineffective, procedural interventions are then considered for the patient.

Ablative procedures such as rhizotomy or mechanical balloon compression aim to disrupt pain transmission by damaging the trigeminal nerve or ganglion. However, these procedures can lead to complications including postoperative dysesthesia, corneal numbness, and anesthesia dolorosa [15]. Radiosurgery involves targeting a lesion near the nerve root with a focused dose of ionizing radiation, which can also interrupt pain signals. The onset of pain relief from radiosurgery is typically delayed, taking weeks or months, and side effects may include facial sensory loss and paresthesias. This non-invasive approach is often more suitable for elderly individuals who may not be able to undergo open surgery [9]. Additionally, neurectomy, which involves cutting the peripheral branches of the trigeminal nerve, is another option, but its effectiveness as a definitive treatment varies [16]. Microvascular decompression stands as the last and often the most definitive treatment option for TN or HFS [17]. This intricate neurosurgical procedure entails performing a craniotomy or craniectomy in the posterior fossa to locate and alleviate the pressure of the offending blood vessels on the trigeminal or facial nerve. During the surgery, Teflon padding is inserted between the nerve and the vessel. This intervention can lead to prolonged pain relief, lasting over 10 years for some patients. Although it is the most effective method, it is also the most invasive. Associated risks include reduced hearing, cerebellar hematoma, CSF leaks, infarction, and facial weakness [18].

Previous use of endovascular stents

The considerable impact of NVCS on patients' lives, coupled with the potential complications associated with open craniotomies, underscores the need for innovative minimally invasive treatment options. In recent years, there has been a significant shift toward endovascular approaches for treating various neurovascular conditions, including aneurysms, strokes, arteriovenous malformations, and arteriovenous fistulas [19]. Recent advancements include the development of various stents, such as flow diverters. While the primary use of intracranial stenting has been in managing intracranial aneurysms, extensive research is also exploring its application in treating patients with intracranial atherosclerosis [6]. The Neuroform (Stryker, Kalamazoo, MI) stent is a commonly used stent. It features an open-cell design made from a nitinol tubular mesh arranged in a "zig-zag" pattern [20]. This stent is offered in various diameters and lengths, providing enhanced adaptability in placement. Flow diverters, on the other hand, are dense-mesh stents placed at the neck of intracranial aneurysms within the parent vessel. Their role is to redirect blood flow away from the aneurysm and back into the parent artery, thereby reducing aneurysmal blood flow and eventually leading to aneurysm obliteration. Over time, these stents promote neo-intimal growth on the parent vessel, aiding in the restoration of normal vascular anatomy [21]. The Pipeline (Medtronic, Minneapolis, MN) device, an FDA-approved flow-diversion device, is utilized for managing intracranial aneurysms [22]. It gained approval in the United States in 2011 following the Pipeline for Uncoilable and Failed Aneurysms trial, which showed that flow-diversion with the Pipeline device was safe (low risk of stroke or death) and effective (high rate of complete aneurysm obliteration without flow-limiting stenosis) for treating large unruptured intracranial aneurysms that had failed previous endovascular treatments [23]. The device features a self-expanding mechanism and is made from a blend of three-quarters chromium-cobalt and one-quarter platinum-tungsten alloy. Like other flow diverters, it covers about 30-35% of the arterial wall surface area and has a porosity of roughly 65-75%. A newer version, the Pipeline Flex Embolization Device (PED Flex; Medtronic, Minneapolis, MN), approved by the FDA in 2015, offers the added advantage of being resheathable [24]. Neurointerventionalists have additionally been improving techniques of stenting and commonly access smaller vessels than previously for a multitude of techniques, including the SCA in thrombectomy and stenting of SCA aneurysms [25,26].

## Materials and methods

**Figure 1:**
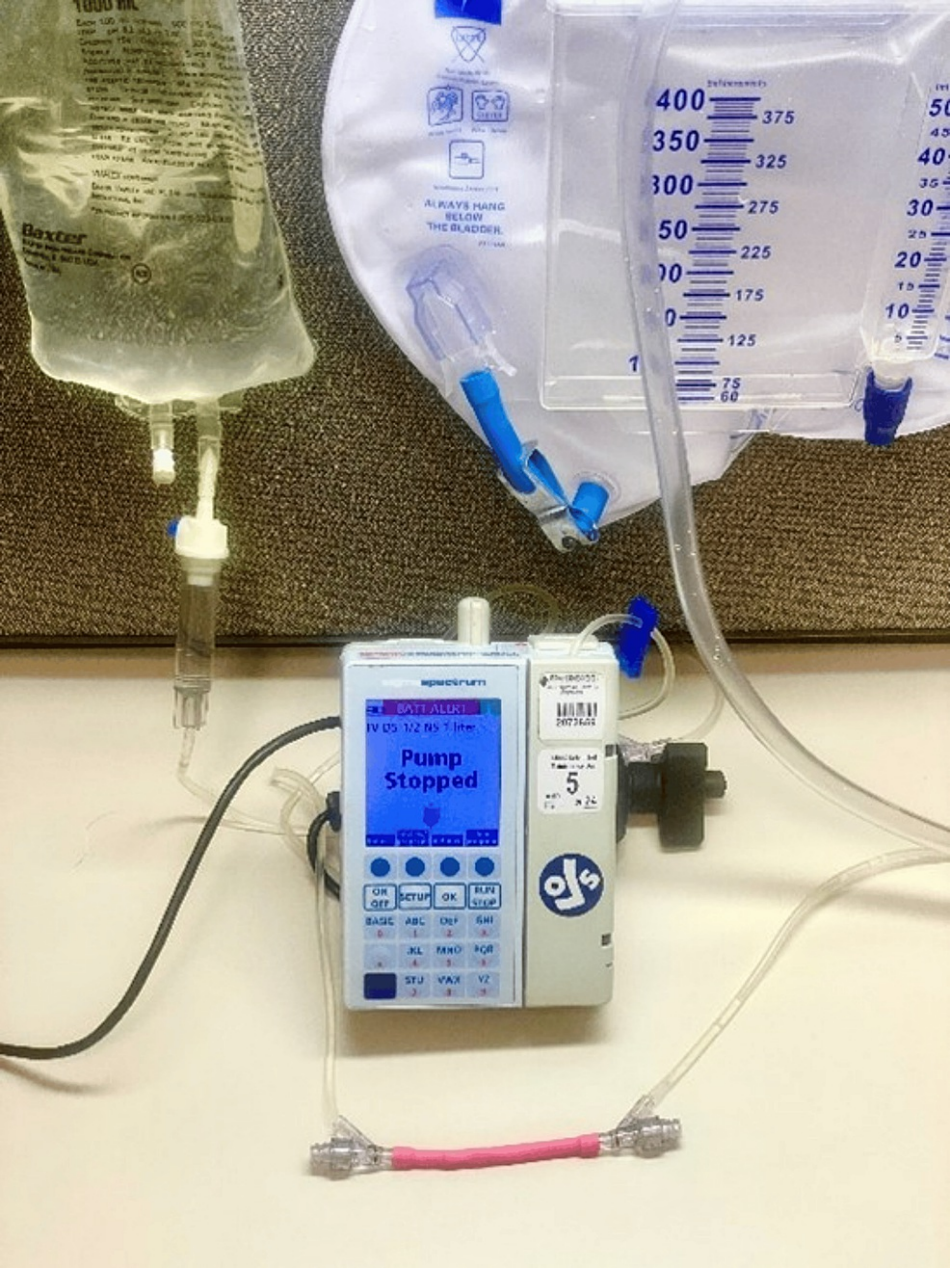
Blood vessel model In vitro blood vessel model featuring IV tubing attached at both ends, with the distal end linked to a reservoir. The IV pump was calibrated to simulate cardiac output, delivering 60 ml per minute. ml: milliliter, IV: intravenous

The study utilized rubber tubing (NUOLUX, Guangdong, China) to simulate blood vessels with dimensions of 5 mm in diameter and 80 mm in length to investigate the effects of endovascular stents on pulsatile flow. As seen in Figure 1, an IV pump filled with saline was employed to propel fluid through the tubing, simulating the pulsatile flow characteristic of cardiac output at 60 ml per minute. IV tubing was connected at both the proximal and distal ends of the tubing to ensure a continuous flow circuit, with a drainage bag attached to serve as a fluid reservoir. Within this experimental framework, three distinct stents were deployed into the rubber tubing to assess their impact on flow dynamics: a Pipeline Flex Embolization Device (Medtronic, Minneapolis, MN) measuring 5x35 mm and Neuroform Atlas Stents (Stryker, Kalamazoo, MI) in two sizes: 4.5x30 mm and 4.0x24 mm. To establish a baseline for pulsatile flow, the rubber tubing was initially tested without any stent in place. A micro-Doppler device confirmed the presence of a signal, indicative of flow within the tubing. Subsequently, a SonoSite X-Porte ultrasound (Fujifilm, Tokyo, Japan) unit in Doppler mode was utilized to record baseline waveforms by placing the probe directly over the rubber tubing, capturing the flow dynamics before stent deployment. Following the deployment of each stent, the micro-Doppler was employed once more to ascertain signal detection, signifying the maintenance of flow post-intervention. The ultrasound unit was then used to obtain Doppler waveforms at three locations relative to each stent: proximal, directly over, and distal to the stent. This approach facilitated a comprehensive assessment of the stents' effects on flow pulsatility across these distinct regions. The study's primary objective was to evaluate and compare the pulsatility measures between the different stents. To achieve this, quantitative measures of pulsatility were extracted from the Doppler waveforms, focusing on parameters such as peak systolic velocity, end-diastolic velocity (EDV), and pulsatility index (PI). These measures were calculated using tools within the ultrasound unit. Statistical analyses were applied to these data to discern any significant differences in pulsatility attributable to the stent types. The statistical methods included ANOVA to compare the pulsatility measures across the different stent conditions. A p-value of less than 0.05 was considered statistically significant, indicating a meaningful difference in flow dynamics attributable to the stent deployment.

## Results

This study aimed to evaluate the hemodynamic changes induced by the implantation of different neurovascular stents in a blood vessel model. The primary variables of interest were the peak systolic velocity (Vmax), PI, and EDV measured at various points relative to the stent placement, as shown in Tables 1-3. Baseline characteristics were established in the absence of a stent, providing a control for comparative analysis. At baseline, without any stent placement, the mean Vmax observed was 43.52 cm/s, ranging from 36.9 to 50.3 cm/s. The PI had a mean value of 2.428, with a range from 1.84 to 2.71. The mean EDV was recorded at 2.26, with a range spanning from 1.78 to 3.12. The introduction of neurovascular stents resulted in notable changes in hemodynamic parameters. These changes varied depending on the stent type and the measurement location relative to the stent (proximal, over, and distal).

**Table 1:** Comparison of PI over stented areas across different stents versus no stent control PI: pulsatility index, mm: millimeter

	No stent	Pipeline 5x35 mm	Atlas 4.5x30 mm	Atlas 4x24 mm
Recording 1	48.1	11.6	15.6	14.7
Recording 2	50.3	12.5	16.9	13.4
Recording 3	36.9	12.9	16	14.2
Recording 4	41.8	13.8	13.4	12.5
Recording 5	40.5	15.6	15.6	14.2
Mean	43.52	13.28	15.5	13.8

**Table 2:** Comparison of Vmax (cm/s) over stented areas across different stents versus no stent control The data was represented as cm/s. cm: centimeter, s: second, mm: millimeter, Vmax: maximum velocity

	No stent	Pipeline 5x35 mm	Atlas 4.5x30 mm	Atlas 4x24 mm
Recording 1	48.1	11.6	15.6	14.7
Recording 2	50.3	12.5	16.9	13.4
Recording 3	36.9	12.9	16	14.2
Recording 4	41.8	13.8	13.4	12.5
Recording 5	40.5	15.6	15.6	14.2
Mean	43.52	13.28	15.5	13.8

**Table 3:** Comparison of EDV (cm/s) over stented areas across different stents versus no stent control The data was represented as cm/s. cm: centimeter, s: second, mm: millimeter, EDV: end-diastolic velocity

	No stent	Pipeline 5x35 mm	Atlas 4.5x30 mm	Atlas 4x24 mm
Recording 1	1.78	1.78	0.89	0.89
Recording 2	1.78	1.34	0.89	0.89
Recording 3	1.78	2.23	0.89	0.89
Recording 4	3.12	2.23	0.89	0.89
Recording 5	2.67	1.78	1.34	0.45
Mean	2.226	1.872	0.98	0.802

Pipeline 5x35 mm stent

Proximal to the Pipeline stent, the Vmax mean decreased to 31.22, with a PI mean of 2.412 and an EDV mean of 1.426. Over the stent, Vmax further decreased to 13.28, with significantly lower PI and EDV values. Distally, Vmax and PI values showed moderate decreases, indicating a reduction in blood flow velocity and pulsatility beyond the stent.

Atlas 4.5x30 mm stent

Measurements proximal to this stent showed a Vmax mean of 31.44, with a PI mean of 2.112 and an EDV mean of 1.16. Over the stent, reductions in all parameters were observed, with a Vmax mean of 15.5. Distally, the Vmax increased to 34.48, still lower than baseline, with corresponding decreases in PI and EDV.

Atlas 4x24 mm stent

Proximal measurements revealed a Vmax mean of 31.16, a PI mean of 2.096, and an EDV mean of 1.1496. Over the stent, there was a significant reduction in Vmax to 13.8. Distally, the Vmax was measured at 25.64, with PI and EDV values also reduced from baseline.

Changes in pulsatile flow characteristics

**Figure 2:**
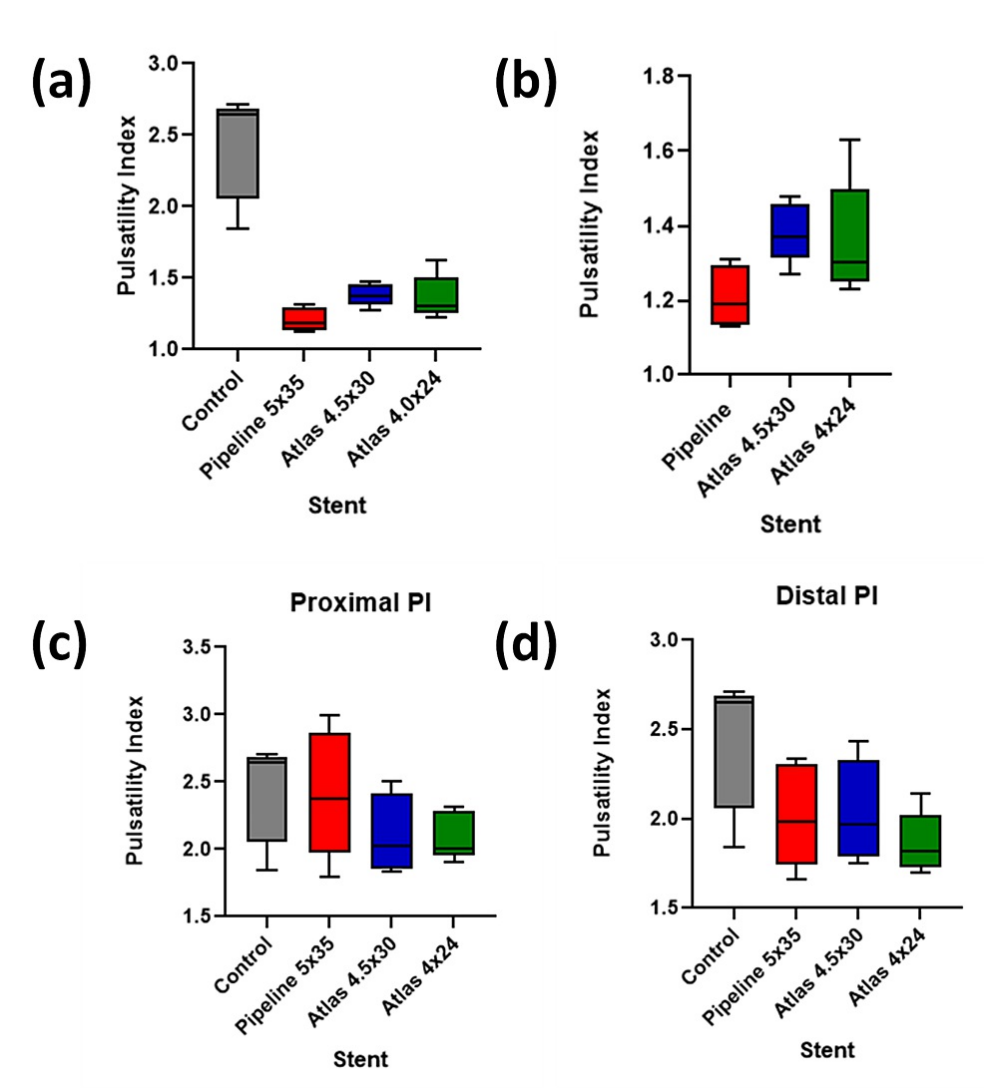
Evaluation of PI across different stents versus control Figure 2a demonstrates a reduction in PI over the stented area in all stents compared to control (P < 0.0001). Figure 2b compares PI over the stented area, which did not reach statistical significance (P = 0.0652). Figure 2c compares PI proximal to the stents, which did not differ significantly from the control (P = 0.2777). Figure 2d compares PI distal to stents, which was significantly lower in stent groups versus control (P = 0.0416). PI: pulsatility index

**Figure 3:**
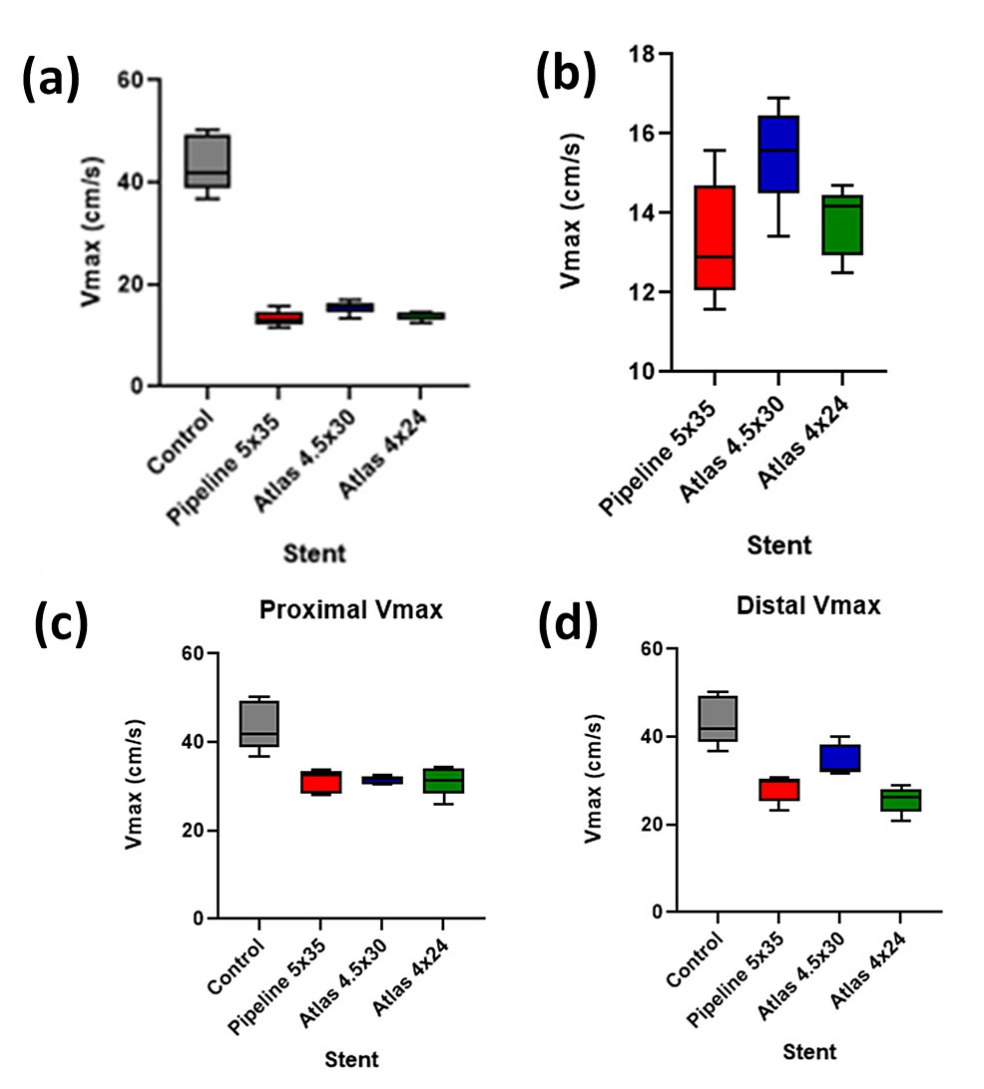
Evaluation of Vmax (cm/s) across different stents versus control Figure 3a shows a significant reduction in Vmax over the stented area versus the control (P < 0.0001). Figure 3b shows a significant difference in Vmax over stented areas when comparing the stents to each other (P < 0.05). Figure 3c compares the proximal Vmax values compared to the control (P < 0.0001). Figure 3d compares the distal Vmax values compared to the control (P < 0.0001). Vmax: maximum velocity, cm: centimeter, s: second

**Figure 4:**
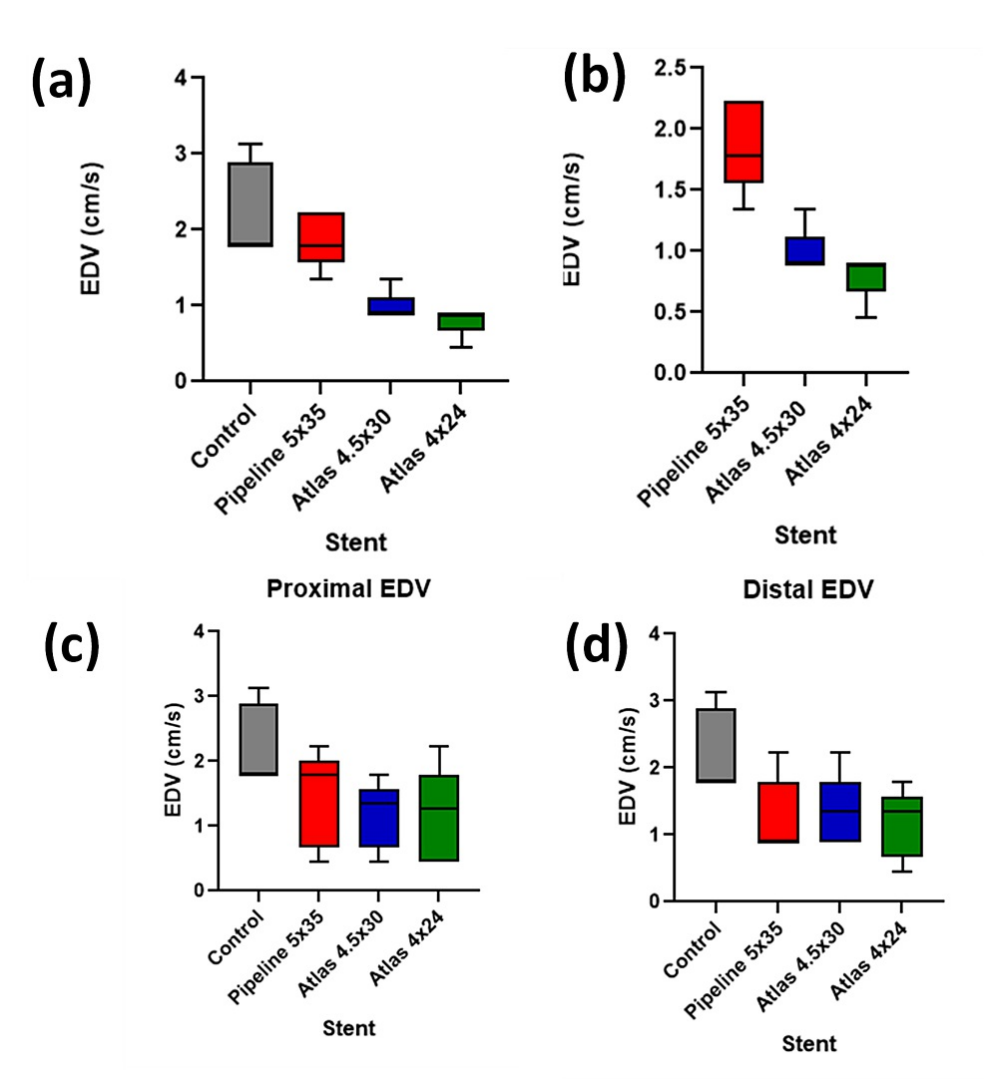
Evaluation of EDV (cm/s) across different stents versus control Figure 4a shows a significant reduction in EDV over the stented area versus the control (P < 0.0001). Figure 3b shows a significant difference in EDV over stented areas when comparing the stents to each other (P <0.0001). Figure 4c compares the proximal EDV values to the control and is not statistically significant (P < 0.0641). Figure 4d compares the distal EDV values compared to the control (P < 0.05). EDV: end-diastolic velocity, cm: centimeter, s: second

The PI of the Pipeline 5x35 mm stent was the lowest among the studied stents, followed by the Atlas 4x24 mm and Atlas 4.5x30 mm stents, as shown in Figure 2a-2d. These reductions were statistically significant compared to the control (P < 0.0001), although differences among the stents did not reach statistical significance (P = 0.0652). For Vmax, seen in Figure 3a-3d, the Pipeline stent showed the lowest value, significantly lower than the control (P < 0.0001). The comparison between the stents also indicated significant differences (P < 0.05). EDV comparisons demonstrated in Figure 4a-4d showed a significant reduction from control across all stent placements (P < 0.0001). However, the proximal PI means for the stents were not significantly lower than the control (P = 0.2777), whereas the proximal Vmax values were significantly lower (P < 0.0001). Distal measurements showed the Atlas 4x24 mm stent had the lowest PI and Vmax values, significantly lower than the control for both PI (P = 0.0416) and Vmax (P < 0.0001). Distal EDV values also showed significant reductions from the control across all stent types (P = 0.0319). These findings demonstrate the effectiveness of neurovascular stents in altering hemodynamic parameters, with significant reductions in Vmax, PI, and EDV observed post-implantation.

## Discussion

The exploration into the efficacy of various neurovascular stents within an in vitro blood vessel model, aimed at reducing pulsatility to aid patients suffering from NVCS like TN or HFS, presents novel insights into potential minimally invasive therapeutic strategies. This study's findings highlight significant alterations in hemodynamic parameters (Vmax, PI, and EDV) following the deployment of Pipeline, Atlas 4.5x30 mm, and Atlas 4x24 mm stents. These modifications indicate the stents' capacity to modulate arterial pulsatility. However, the intrinsic neural reactions at the REZ remain unknown given the current study of intraluminal hydrodynamic changes of an inert material.

Hemodynamic modulation by stenting

**Figure 5:**
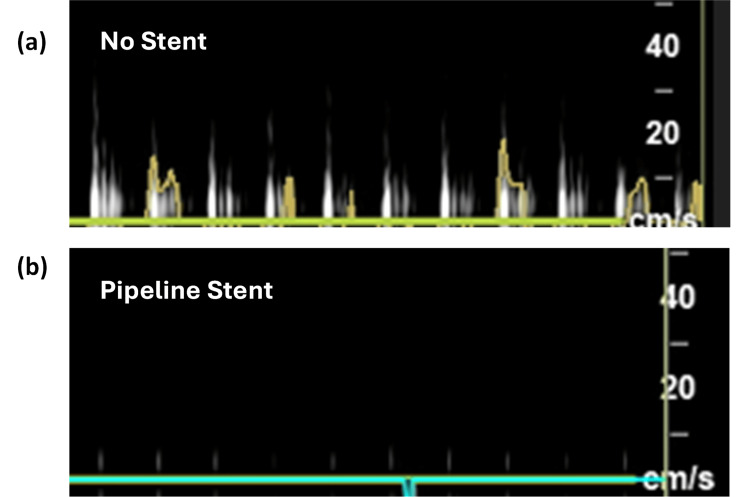
Doppler flow waveforms comparing pulsatility with no stent versus Pipeline stent Doppler flow characteristics in the blood vessel model after deploying the Pipeline stent (Figure 5b) versus no stent control (Figure 5a). A reduction in Vmax, PI, and EDV is seen, with near resolution of pulsatile flow. Waveforms were obtained by utilizing the ultrasound in Doppler mode. All recordings in this figure were taken directly over the targeted stented area. The y-axis represents velocity in cm/s. cm: centimeter, mm: millimeter

**Figure 6:**
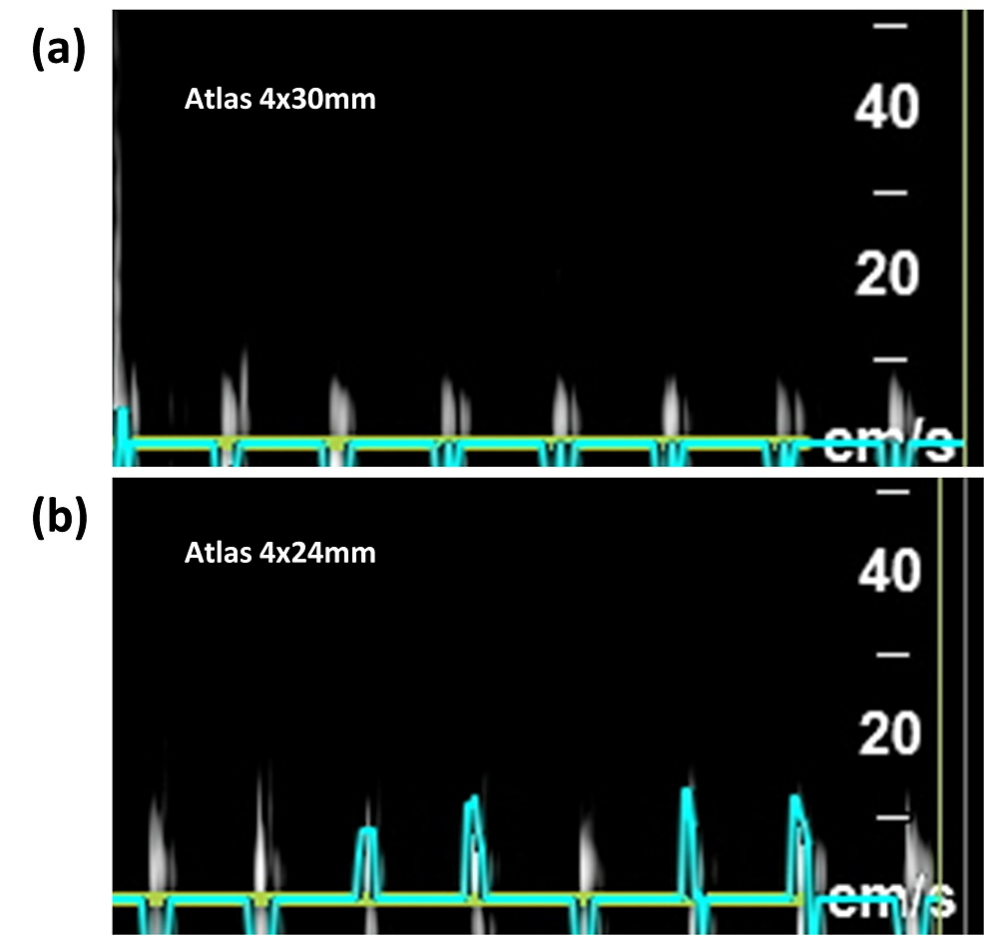
Doppler flow waveforms comparing pulsatility in Atlas stents Doppler flow characteristics in the blood vessel model after deploying the Atlas 4x30 mm stent (Figure 6a) versus the Atlas 4x24 mm stent (Figure 6b). A reduction in Vmax, PI, and EDV is seen, with a modest reduction in pulsatile flow across the stented area. Waveforms obtained utilizing the ultrasound in Doppler mode demonstrate pulsatile flow characteristics over the blood vessel model. All recordings in this figure were taken directly over the area of the stents. The Y-axis represents velocity in cm/s. mm: millimeter, cm: centimeter

The observed decrease in Vmax, particularly pronounced over the stented sections, underscores the stents' role in reducing blood flow velocity. This reduction, as seen in Figure 5a-5d is critical in mitigating the pulsatile force exerted on cranial nerves, which is implicated in the pathophysiology of NVCS. Notably, the Pipeline stent in Figure 5b showed the most substantial reduction in Vmax and pulsatility, suggesting its potential superiority in achieving targeted flow modification. Conversely, the Atlas stents, with their varying sizes and slightly different hemodynamic impacts, present a nuanced option for tailoring treatment based on the specific vascular anatomy and pathology of each patient (Figure 6a-6b). The absence of significant differences among the stents concerning PI reduction suggests a common underlying mechanism regarding their impact on vessel compliance and resistance, despite the disparities in design and material. This observation could guide clinical stent selection, emphasizing the necessity of considering individual patients. Moreover, the significant reduction in PI across all stent types further supports the hypothesis that altering vascular dynamics can lead to decreased nerve compression. This finding aligns with the theoretical underpinning of microvascular decompression surgery, which aims to eliminate the aberrant pulsatile compression of cranial nerves. However, the deployment of stents offers a less invasive approach, with the potential to minimize the risks associated with open cranial surgery if they can be deployed safely.

Several case reports in recent literature have demonstrated significant improvements in both TN and HFS using endovascular devices [27,28]. One notable case involved a patient with secondary TN linked to an SCA aneurysm who was treated with stent-assisted coiling using a 2x12 mm Leo baby braided stent (Balt Group, Montmorency, France). Remarkably, the patient experienced complete relief from neuralgic pain within the first 24 hours post-treatment, a relief that persisted during follow-up. At the nine-month mark, the stent remained patent. This immediate pain remission post-endovascular intervention, long before any remyelinating process could occur, highlights the significant role of arterial pulsation in axon hyperexcitability and the development of ectopic activity [28]. Another case reported by Miyazaki et al. involved a patient with HFS who had a left VA dissecting aneurysm. Interestingly, the aneurysm was not in contact with the facial nerve's root entry zone, but a distal VA trunk likely caused the compression. The patient underwent stent-assisted coiling with an Enterprise-2 vascular reconstruction device stent (J&J MedTech, Irvine, CA), one Orbit Galaxy complex coil (Codman Neurovascular, Raynham, MA), and nine Target 360 nano coils (Stryker, Kalamazoo, MI) under general anesthesia. The stent, placed in the parent artery, covered both the aneurysmal neck and the distal VA trunk, the presumed area impacting the facial nerve. Post-procedure, there was a substantial reduction in HFS, with a 50% improvement noted at one month and complete resolution at one year, with no recurrence at 32 months. A follow-up MRI showed no positional change between the offending vessel and the facial nerve REZ but indicated some new endothelialization on the stent surface [27].

The immediate improvement in HFS following stent placement is thought to be influenced by factors such as the stent reducing normal arterial pulsation in the causative vessel wall. This effect is due to the stent's rigidity and added thickness. Additionally, subtle positional adjustments in the causative vessel may occur due to the straightening effects of the stent. Over time, the endothelialization of the stent's inner surface might progress, gradually lessening the pulsatile force against the root entry zone, which could account for the gradual improvements observed in facial spasms. Arterial straightening is a common occurrence post-intracranial stenting, with changes in the angle of the stented artery reported to continue between 6 and 34 months following stent deployment [27,28]. Furthermore, minimal movement of the distal VA may occur after stent placement, though significant positional changes in the responsible VA area are not typically evident in follow-up angiograms. A study by Chun et al. introduced a novel intra-arterial neurovascular decompressor as a potential treatment for NVCS. This device, a modified Neuroform Atlas stent, was found to be safely applicable to intracranial arteries and is being tested for its efficacy in altering the course of an artery compressing a cranial nerve [29]. In other cases reported by Santiago-Dieppa et al. and Nagashima et al., relief of HFS was observed following endovascular aneurysm treatment, even without changes in the size of the aneurysm in contact with the REZ of the facial nerve at the time symptoms ceased [7,30]. This suggests that a reduction in pulsatility may be more crucial for symptom improvement than decreasing compression in the nerve's REZ.

Clinical implications, limitations, and future directions

The current model employed in this study is a non-biological tube, restricting the evaluation of hydrodynamics to an inert material. Consequently, the intricate interplay of vascular resistance, vasoreactivity, and multifactorial conditions that influence hemodynamics in vivo is not fully captured. It is essential to recognize that the arterial system and its hemodynamics are subject to various physiological influences that extend beyond the scope of this experimental setup. Moreover, this study predominantly focuses on the vascular parameters' reaction to compression, neglecting the neural aspects of intrinsic reactions within the nervous system. While the examination of vascular dynamics is crucial, a comprehensive understanding of microvascular compression necessitates the consideration of broader parameters beyond mere flow dynamics.

Furthermore, the disparity in size between the stent and tubing utilized in this study and the smaller dimensions of arteries such as the AICA and SCA suggest potential limitations in extrapolating the findings to distal compression scenarios. Additionally, it is important to acknowledge that endovascular techniques, while promising, can introduce complications such as vessel damage during selection, vessel perforation during deployment, vessel occlusion, and distal stroke. These complications underscore the challenges associated with translating experimental results into clinical practice. Despite these limitations, the simulation with the synthetic model provides valuable insights that could serve as a foundation for future research. While the model may not fully replicate the complexities of living systems, the derived values offer a starting point for further investigation. Subsequent studies in living models and human subjects are warranted to validate these findings and explore the potential applications in the clinical management of microvascular compression pathologies. Additionally, future investigations should aim to elucidate the optimal stent characteristics (e.g., size, material, and design) for maximally reducing pulsatility without compromising vessel integrity or blood flow to critical brain regions. Exploring the biological response to stenting, including endothelialization and potential inflammatory reactions, will also be crucial for optimizing outcomes and minimizing complications.

## Conclusions

This study contributes valuable data to the burgeoning field of endovascular treatment for NVCS, suggesting that stenting could represent a less invasive, effective strategy for reducing arterial pulsatility and alleviating the symptoms of conditions like TN and HFS. Importantly, the findings of this experimental study demonstrate that the hydrodynamics within the simulation with this synthetic model resemble values that can be the basis for future research in living models and subsequently be tested in humans for this type of pathology. This specific change underscores the precision with which endovascular interventions can target and mitigate the hemodynamic factors contributing to NVCS without affecting the overall blood flow. These findings underscore the need for continued innovation in endovascular technology and a multidisciplinary approach to treating these complex disorders. The ability to fine-tune the intervention to address specific hemodynamic disturbances offers hope for improved patient outcomes and quality of life, marking a significant step forward in the treatment of these challenging conditions. Future studies will be done to observe the changes in the hemodynamics of a normal cerebral arterial vessel that was modified by an endoprosthesis and its possible repercussions on a modified endothelium.
